# S12-2 The effectiveness of a peer-led school-based walking intervention on adolescent girls’ physical activity: the Walking In ScHools (WISH) study

**DOI:** 10.1093/eurpub/ckad133.059

**Published:** 2023-09-11

**Authors:** Angela Carlin, S Maria O'Kane, Leanne C Doherty, Alison M Gallagher, Ian M Lahart, Russell Jago, Gary McDermott, Maria Faulkner, Marie H Murphy

**Affiliations:** Centre for Exercise Medicine, Physical Activity and Health, Sports and Exercise Sciences Research Institute, Ulster University, Belfast Campus, Belfast BT15 1ED, UK; Centre for Exercise Medicine, Physical Activity and Health, Sports and Exercise Sciences Research Institute, Ulster University, Belfast Campus, Belfast BT15 1ED, UK; Centre for Exercise Medicine, Physical Activity and Health, Sports and Exercise Sciences Research Institute, Ulster University, Belfast Campus, Belfast BT15 1ED, UK; Nutrition Innovation Centre for Food and Health (NICHE), Biomedical Sciences Research Institute, Ulster University, Coleraine Campus, Coleraine BT52 1SA, UK; Doctoral College, Ulster University, Belfast Campus, Belfast BT15 1ED, UK; Faculty of Education, Health and Wellbeing, University of Wolverhampton, Walsall Campus, Walsall WS1 3BD, UK; Centre for Exercise, Nutrition & Health Sciences, School for Policy Studies, University of Bristol, Bristol BS8 1TZ, UK; Centre for Exercise Medicine, Physical Activity and Health, Sports and Exercise Sciences Research Institute, Ulster University, Belfast Campus, Belfast BT15 1ED, UK; Department of Law and Humanities, Atlantic Technological University, Letterkenny Campus, Letterkenny, Ireland; Centre for Exercise Medicine, Physical Activity and Health, Sports and Exercise Sciences Research Institute, Ulster University, Belfast Campus, Belfast BT15 1ED, UK; Physical Activity for Health Research Centre (PHARC), Institute for Sport, Physical Educ

## Abstract

**Purpose:**

Adolescent girls are failing to meet current physical activity (PA) guidelines. PA behaviours track from childhood to adulthood so providing adolescent girls with opportunities to be physically active may have benefits well beyond childhood. The effects of walking interventions on adult health are known, however less is understood about the potential of walking to promote PA in adolescents. Following the WISH pilot feasibility study that reported an increase in light intensity PA, a full trial has been undertaken to evaluate the effectiveness of a novel school-based walking intervention at increasing PA levels of adolescent girls.

**Methods:**

The WISH study is a clustered randomised controlled trial in which a peer-led, school-based, brisk walking intervention is compared to usual PA in adolescent girls (12-14years). Data was collected at four timepoints over a 13-month period. Following baseline data collection, schools were randomly allocated to intervention (n = 9) or control (n = 9). In intervention schools, older pupils (aged 15-18years) were trained as walk leaders and led younger girls in 10-15min walks before school and at break-times across the school year (20-22weeks). The primary outcome was accelerometer-measured total PA (post-intervention). Secondary outcomes included anthropometry, quality of life and sleep.

**Results:**

In total, 590 participants (mean(SD) age 12.6(0.6)years) were recruited from 18 schools across Northern Ireland (n = 9) and the Republic of Ireland (n = 9). Within the intervention schools, 149 walk leaders were trained (mean(SD) age 17.1(0.8)years). At baseline (n = 535), mean(SD) time spent in moderate to vigorous PA (MVPA) was 39.2(17.1)mins/day and only 66 (12%) girls achieved PA guidelines of 60minutes MVPA per day. Post-intervention interviews were conducted with n = 18 walk leaders and n = 9 teachers. Focus groups (n = 9) were conducted with a mix of high and low attenders. Data analysis is ongoing, and the process evaluation is underway (both will be complete by May 2023).

**Conclusion:**

To our knowledge, this is the first fully powered trial to investigate the effectiveness of a peer-led brisk walking intervention in adolescent girls. The current study builds on a promising pilot trial which confirmed the feasibility of the intervention. If the intervention increases PA, it has potential to be implemented widely.

